# Monthly Record of Deaths in the City of Buffalo

**Published:** 1854-05

**Authors:** Jas. M. Newman

**Affiliations:** Health Physician


ART. VIII.—*Record of Mortality in the City of Buffalo, for the Month of March*, 1854. By J. M. NEWMAN, Health Physician.

| DISEASES. | No. | Males. | Females. |
|---|---|---|---|
| Accidents, | 1 | 1 | |
| Apoplexia, | 4 | 1 | 3 |
| " Pulmonalis, | 1 | 1 | |
| Asphyxia Immersorum, | 4 | 2 | 1 |
| Asthma, | 1 | 1 | |
| Cirnhosis, | 1 | 1 | |
| Coma, | 1 | 1 | |
| Cyanosis, | 1 | | 1 |
| Cynanche Trachealis, | 10 | 6 | 4 |
| Eclampsia, | 10 | 3 | 7 |
| Enteritis, | 2 | 2 | |
| Febris Typhus, | 2 | 2 | |
| " Typhoid, | 3 | 1 | 2 |
| " Puerperarum, | 2 | | 2 |
| " Scarlatina, | 6 | 5 | 1 |
| Hæmorrhagia Hepatis, | 1 | 1 | |
| Hepatitis Chronica, | 1 | | 1 |
| " Induratio, | 1 | | 1 |
| Hydrophobia, | 2 | 1 | 1 |
| Hydrops, | 2 | 2 | |

REGISTER OF MORTALITY—(Continued.)

| DISEASES. | No. | Males. | Females. |
|---|---|---|---|
| Hydrops Celluaris totius corporis, | 1 | 1 | |
| " Cerebri, | 5 | 2 | 2 |
| " Pectoris. | 2 | 1 | 1 |
| " Pericardii, | 1 | 1 | |
| Hyperæmia Cerebralis, | 3 | | 2 |
| " Pulmonalis, | 2 | | 1 |
| Inflam. Nervi Vagi, | 1 | | 1 |
| Laryngitis, | 2 | 1 | 1 |
| Marasmus, | 2 | 1 | 1 |
| Negligentia, | 4 | 3 | |
| Paralysis Pulmonalis, | 2 | 2 | |
| Parturitio, | 1 | | 1 |
| Pertussis, | 1 | | |
| Phlebitis, | 1 | 1 | |
| Phthisis Pulmonalis, | 31 | 10 | 20 |
| Phrenitis, | 2 | 1 | 1 |
| Pneumonia, | 9 | 3 | 4 |
| " Typhoides, | 2 | 1 | 1 |
| Rubeola, | 7 | 5 | 2 |
| Scalded, | 1 | 1 | |
| Scrofula, | 1 | | 1 |
| Senectus, | 1 | 1 | |
| Spasmus Glottidis, | 1 | | 1 |
| Still-born, | 6 | | |
| Strangulated Hernia, | 1 | | 1 |
| Tupis Catharshalis, | 1 | 1 | |
| Ignotus, | 34 | 16 | 16 |
| | 181 | | |

| | |
|---|---|
| Males, | 83 |
| Females, | 81 |
| Sex not given, | 17 |
| Total, | 181 |

AGES.

| | | | |
|---|---|---|---|
| Still-born, | 6 | Between 6 " " 12 " | 10 |
| 1 day, | 2 | " 12 " " 20 " | 8 |
| Between 1 day and 15 days, | 7 | " 20 " " 30 " | 14 |
| " 15 days and 30 days, | 5 | " 30 " " 40 " | 15 |
| " 1 month and 3 months, | 6 | " 40 " " 50 " | 15 |
| " 3 months and 6 months, | 13 | " 50 " " 60 " | 7 |
| " 6 " " 9 " | 5 | " 60 " " 70 " | 5 |
| " 9 " " 12 " | 8 | " 70 " " 80 " | 2 |
| " 1 year and 3 years, | 32 | Ages not given, | 2 |
| " 3 years and 6 years, | 23 | | |
| Total, | | | 181 |

NATIVITY.

| | | | |
|---|---|---|---|
| American, | 54 | French, | 4 |
| English, | 4 | Swiss, | 1 |
| Scotch, | 2 | New Brunswick, | 1 |
| Irish, | 12 | Colored, | 3 |
| German, | 58 | Country not given, | 42 |
| Total, | | | 181 |

